# Impact of *Salix mucronata* bark and betaine as natural alleviators on semen quality, serum biochemistry, and oxidative capacity in heat-stressed rabbit bucks

**DOI:** 10.1007/s11250-025-04781-1

**Published:** 2025-12-24

**Authors:** Maher F. M. Farag, Ahmed H. Daader, Abdel-Halim A. El-Darawany, Lila B. Bahgat, Khaled M. Al-Marakby

**Affiliations:** Animal Production Department, Faculty of Agriculture, Zagazig University, Zagazig, Egypt

**Keywords:** Heat stress, Willow bark, Betaine, Rabbit buck, Semen quality, Oxidative capacity

## Abstract

The study aimed to assess the mitigation of negative impacts of heat stress (HS) by the willow bark (*Salix mucronata* Thunb.) powder (SWBP) or betaine (BET) as natural antioxidants on semen quality, blood biochemicals, and oxidative status in rabbit bucks. Under control conditions, forty mature NZW adult males were distributed into five groups of eight replicates. The 1st group fed the basal diet without any additives (control), whereas the other groups were fed the basal diet contained 10 g of SWBP (SWBP_10_), 20 g of SWBP (SWBP_20_), 1 g of BET (BET_1_), or 2 g of BET (BET_2_) per Kg of diet. The results demonstrated that supplementing SWBP significantly reduced (*P* < 0.001) the respiration rates (RR), as well of ear (ET) and rectal temperatures (RT), while BET_2_ recorded the lowest (*P* = 0.004) skin temperature (ST). All supplements markedly improved serum total protein, total lipid, and the activities of the AST and ALT (*P* < 0.001). Moreover, SWBP_20_ and BET_2_ enhanced serum albumin and globulin levels (*P* ≤ 0.01), superoxide dismutase, and total antioxidant capacity (*P* < 0.001). Both SWBP and BET also elevated testosterone levels and shortened reaction time (*P* < 0.001). SWBP_20_ showed the highest testosterone and shortest reaction time. Semen quality parameters including pH, ejaculate volume, motility, sperm concentration, and viability were significantly improved (*P* < 0.05). In seminal plasms, both supplements increased glutathione and TAC levels while decreasing malondialdehyde (*P* < 0.001). The findings indicate that dietary SWBP and BET could improve semen quality, blood parameters, and oxidative capacity of rabbit bucks under HS conditions. Therefore, SWBP_20_ or BET_2_ could be used as HS alleviators for New Zealand White bucks during the hot Egyptian summer months by regulating the antioxidant/oxidative pathways to support reproductive health.

## Introduction

In developing countries, rabbits serve a crucial role in providing animal protein. Nevertheless, heat stress (HS) represents the primary constraint on rabbit production in subtropical areas such as Lower Egypt (Daader et al. 2016). HS is defined as a non-specific physiological response that occurs when the body is exposed to elevated ambient temperatures, resulting in a redox imbalance and subsequent oxidative stress (OS) detrimental to animal health and performance (Hu et al. 2019). Rabbits struggle in hot climates, a challenge intensified by global warming (Oladimeji et al. 2022). In rabbits, HS triggers an extreme generation of reactive oxygen species (ROS), leading to OS (Jimoh et al. 2017; Bai et al. 2022). This OS, in turn, deteriorates rabbit performance, causes serious health issues, and results in biological damage (Abdel-Khalek 2013). To counteract the detrimental effects of HS in rabbits, numerous nutritional and physiological techniques have been explored to maintain homeostasis or prevent nutrient deficiencies (Sultana et al. 2022). Specifically, adding functional active substances to the diet can effectively reduce the harmful impacts of HS in rabbits (Liang et al. 2022).

Recent studies show that the antioxidant properties of natural sources of bioactive compounds, such as polyphenols, position them as effective alleviators of HS. *Salix mucronata* Thunb. (safsaf willow), is widely distributed along the Nile River in Egypt. Like other willow trees, extracts from safsaf have also been used in traditional medicine (Beltag et al. 2022). *S. mucronata* contains phenolic compounds, flavonoids, terpenes and lignans. The phenolic compounds isolated, were the most abundant with reported analgesic, antipyretic, anti-inflammatory and anti-rheumatic properties. The therapeutic potential for willow’s natural products may be attributed to their bioactive compounds, which work synergistically to yield the anti-inflammatory effects (Vlachojannis et al. 2011; Nahrstedt et al. 2007). Among various forms, willow bark is an important natural source of salicin, *β*-O-glucoside of saligenin, but also of polyphenols (flavonoids and condensed tannins) with antioxidants, antimicrobial, and anti-inflammatory activity (Saracila et al. 2021). Due to the presence of multiple bioactive compounds in safsaf, which exhibit antioxidant, anti-inflammatory, and antimicrobial activity, we hypothesized that the addition of dietary safsaf can improve reproductive health during hot climates by reducing oxidative stress and enhancing antioxidant capacity.

Betaine (BET) is a natural, stable, and nontoxic substance in living cells. It is endogenously synthesized via choline metabolism or exogenously supplemented to diets as a feed additive. As a methyl-group donor and an osmolyte, BET plays its physiological roles (Arumugam et al. 2021). Betaine is a derivative of the amino acid glycine, with three hydrogen atoms on the nitrogen atom replaced by methyl groups (Gouda et al. 2025). Its unique chemical structure allows it to play various roles such as methyl donation and zwitterionic behavior. Betaine is a quaternary ammonium compound with a positively charged nitrogen atom bonded to four carbon atoms and a negatively charged carboxyl group from its glycine component (Gouda et al. 2025). It serves as a crucial methyl group donor in animal nutrition, synthesized in cellular mitochondria from choline and glycine (Abd El-Ghany and Babazadeh 2022). Betaine’s biological importance extends beyond its role as a methyl donor. Its zwitterionic properties (having both positive and negative charges) enable it to function as a crucial osmolyte (Abdelnour et al. 2021). In this capacity, BET helps maintain cellular water homeostasis and integrity without interfering with the cell’s normal metabolism (Gajardo-Parra et al. 2022; Liu et al. 2024).

BET is a natural anti-heat stress agent for farm animals (Abu Hafsa et al. 2023). The action of BET appears via donation of the methyl group (Cronje 2018), osmoprotectant properties, and anti-inflammatory and antioxidant impacts (Zhao et al. 2018). BET has been found to alleviate HS in rabbits (Daader et al. 2018) and in roosters (Attia et al. 2019). Under HS, the supplementation of BET (1 g/Kg diet) significantly improved the sexual desire, progressive motility, vitality, intact acrosome and membrane integrity, sperm concentration, sperm outputs and fertility in the Animal Production Research Institute (APRI) line rabbit bucks (El-Ratel et al. 2021). During periods of stress, betaine supports normal cellular metabolic function by donating its readily available methyl group for transmethylation reactions. This process leads to the production of vital compounds, such as carnitine, which, in turn, boosts both overall metabolism and the immune system (Abd El-Ghany and Babazadeh 2022). A review of the literature indicates that existing research on the effects of betaine in rabbit diets has exclusively focused on heat stress conditions (Hassan et al. 2011; Chen et al. 2023; Abu Hafsa et al. 2023). However, the role of BET or SWBP in mitigating oxidative stress and improving serum biochemistry, libido, and semen quality of male rabbits during the Egyptian hot summer remains unclear. Therefore, we hypothesize that dietary supplementation of rabbit bucks with dried Safsaf willow (*Salix mucronata* Thunb.) bark powder (SWBP) or betaine (BET) will mitigate oxidative stress and improve serum biochemistry, libido, and semen quality during the Egyptian hot summer.

## Materials and methods

The current study was conducted at the Rabbit Research Farm and Laboratories of the Animal Production Department, Faculty of Agriculture, Zagazig University, Egypt, during the hot summer season, specifically from June to September 2023. This experiment was approved by the Institutional Animal Care and Use Committee (IACUC) of Zagazig University under protocol No. ZU-IACUC/2/F/65/2020.

### Experimental design, animals, management, and diets

Forty sexually mature male New Zealand White (NZW) rabbit, aged between 4 and 4.5 months, with an average body weight of 2750 ± 68.34 g. The rabbits were randomly assigned to five homogenous experimental treatment groups (8 males/each). The first group was fed the basal diet without any additives and served as control, while the other groups received the basal diets supplemented with 10 g of SWBP (SWBP10), 20 g of SWBP (SWBP20), 1 g of BET (BET1), and 2 g of BET2) per Kg of diet.

Each rabbit was housed individually in stainless steel cages measuring 60 × 55 × 40 cm. Throughout the 14-week experimental period, all rabbits were maintained under consistent managerial, hygienic, and environmental conditions. They were acclimatized for one week before the trial commenced. The rabbits were fed *ad libitum* during the experimental period, and fresh water was continuously available to them.

The bark was peeled from the branches and trunks of safsaf willow trees (*Salix mucronata* Thunb.) cultivated on sluiceway banks in Abu Hammad, Sharkia governorate, Egypt. The collected bark was sun-dried, grounded, and kept until using it. Before starting this experiment, the phenolic and flavonoid contents were determined in *Salix mucronata*. BET, a tri-methyl glycine, is a commercial product called Betafin^®^ S4 (Danisco Animal Nutrition, Finland), purchased from Multivita, Sixth of October City, Egypt. The tested feed supplements, SWBP and BET (powder form), were thoroughly mixed with the basal diet (weekly), then packed and kept in the refrigerator. The diet was formulated to meet the nutrient requirements for mature rabbits as recommended by De Blas (2010). Formulation and chemical analysis of the basal diet is presented in Table 1. Nutrients in the basal diet were determined According to AOAC (2000).

**Table 1:** Formulation and chemical composition of the basal diet

Ingredients	%	Chemical composition (on DM basis)	%
Berseem hay (15% CP)	30.00	**Dry matter**	88.76
Wheat bran	32.00	**Organic matter**	90.85
Barley grains	19.00	**Crude protein**	17.75
Soybean meal (44% CP)	14.00	**Ether extract**	2.29
Molasses	3.00	**Crude fiber**	13.36
Limestone	1.30	**Nitrogen free extract**	56.45
Premix*	0.30	**Total Ash**	8.74
Sodium chloride	0.30	DE kcal/kg DM **	2554.90
Anti coccids	0.10		
Total	100		

*: Vitamins and minerals mixture contained: Vit. A 6,000,000 IU, Vit. D3 1,250,000 IU, Vit. E 15,000 mg, Vit. B1 1000 mg, Vit. B6 1000 mg, Vit. B_12_ 6 mg, nicotinic acid 15,000 mg; pantothenic acid 5000 mg, biotin 50 mg, folic acid 500 mg, choline chloride 50% 400 mg, Mn 35 mg, Fe 40 mg, Cu 3.5 mg, Zn 25 mg, iodine 0.25 mg, Se 0.075 mg, cobalt 0.10 mg, and CaCO_3_ up to1000g

**: Digestible energy, evaluated corresponding to (Fekete and Gippert 1986) method as follows:

DE (Kcal/Kg DM) = 4253-[32.6 (CF%) -144.4 (Total ash%)]

### Temperature humidity index

Inside the rabbitry, ambient temperature (AT) and relative humidity (RH) were measured daily at 4 p.m. using a thermo-hygrograph. Then, AT and RH values were used to calculate the temperature-humidity index (THI) based on the equation provided by Marai et al. (2001) as follows:

$${\mathrm{THI}}\,{\text{ = }}\,{\mathrm{d}}{{\mathrm{b}}^ \circ }{\mathrm{C}}\, - \,{\mathrm{[}}\left( {{\mathrm{0}}{\mathrm{.31}}\, - \,{\mathrm{0}}{\mathrm{.31}}\,\left( {{\mathrm{RH/100}}} \right)\, \times \,\left( {{\mathrm{d}}{{\mathrm{b}}^ \circ }{\mathrm{C}}\, - \,{\mathrm{14}}{\mathrm{.4}}} \right)} \right]$$

Where db ^o^C: dry bulb temperature and RH: relative humidity percentage. THI values below 27.8: absence of HS, from 27.8 to 28.9: moderate HS, from 29.0 to 30.0: severe HS, and above 30.0: very severe HS.

**Fig. 1:**
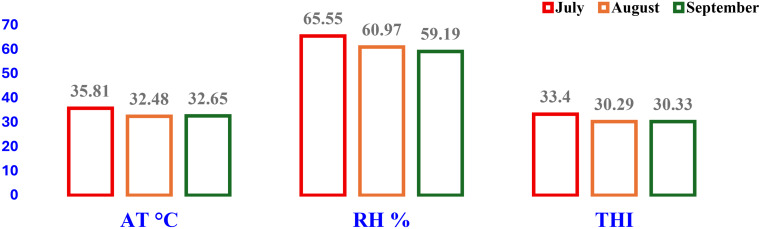
Air temperature (AT °C), relative humidity (RH %), and temperature-humidity index (THI) during the experimental period

The AT, RH, and THI during the trial interval (hot Egyptian summer) are illustrated in Fig. 1. The average values of the THI indicated a state of very severe heat stress.

### Body temperature and respiration rate

The skin temperature (ST), rectal temperature (RT), and ear temperature (ET) were measured individually in all rabbits using a digital thermometer. The ST was assessed at a single location on the body surface, specifically between the loin and the neck. The RT was obtained by inserting the thermometer probe 2 cm deep into the rectum. The ET was measured by placing the thermometer probe in direct contact with the internal central area of the auricle. The measurement durations were minimized to ensure they were taken at similar times. All body temperature measurements were conducted at midday, between 14:00 and 16:00, during the heat stress period. The respiration rate was measured by observing the rabbit’s nostrils and counting their dilations (inhaling) for 60 s using a stopwatch.

### Blood sampling and blood measurements

At the end of the experimental period, blood samples were collected individually from slaughtered rabbits (4 per group) into heparinized and non-heparinized tubes. The heparinized one was to assess the hematological parameters immediately using an automated hematology analyzer (Hospitex Hema Screen 18, Sesto Fiorentino, Italy) following the method declared by Moore et al. (2015). Also, plasma was separated from the heparinized samples by centrifugation at 503× *g* for 20 min and stored at -20 °C until the testosterone hormone assay. The non-heparinized tubes were left to clot, then centrifuged at 503× *g* for 20 min to obtain the serum, which was kept at -20˚C until analyses. Blood serum metabolites, involving total protein (TP), albumin (AL), total lipids (TL), aspartate amino transferase (AST), and alanine amino transferase (ALT) were assessed spectrophotometrically using commercial kits offered by Bio-diagnostic Company (Giza, Egypt), following the manufacturer’s directions. The Globulin level was determined by calculating the differences between TP and AL concentrations. To assess antioxidant profile and OS parameters, the activities of superoxide dismutase (SOD) (Marklund et al. 1982) glutathione peroxidase (GPx) (Flohé and Günzler 1984) and levels of GSH (Anderson 1985) and MDA (Alipour et al. 2006), as well as the TAC (Ghiselli et al. 2000) were determined in rabbit serum using commercial kits and a spectrophotometer (Shimadzu, Kyoto, Japan).

### Semen collection and evaluation

During July, males were trained for adaptation to the experiment conditions and semen collection according to IRRG (2005). Semen ejaculates were collected from the New Zealand White bucks (NZWB), once a week from each buck, using an artificial vagina at 45 °C in the existence of a female. During August and September, 56 semen ejaculates were collected weekly from each rabbit group. As an indicator of libido in NZWB, reaction time, the time elapsed from the introduction of the female to the male’s cage till ejaculation was detected in seconds using a stopwatch. The net ejaculate volume (EV), pH value, mass motility (MM), progressive motility (PM), sperm concentration (SC), live (LS), dead (DS), and abnormal spermatozoa (AS) were assessed.

The semen was maintained in a water bath (37 °C) and then transferred to the laboratory for semen evaluation. Special attention was given to protecting semen from cold or heat shocks and direct light. Throughout semen collection, the place and time of collection, and the collector were kept constant. The ejaculate volume for each buck was recorded after removal of the gel mass and semen pH value was immediately determined using pH paper (p^H^ 0 to 14 universal indicator, Merck). Mass motility was determined using one drop of fresh semen, which was placed on a warmed slide. Mass motility from at least three fields was examined under a light microscope, at 10 ×, and assessed from 0 to 5. At 40× magnification, the percentage of sperm motility was evaluated in several microscopic fields for each semen sample by visual examination using a light microscope with classifications of subjective assessments ranging from 0% to 100% (Theau-Clément et al. 2005; Abo-Elsoud et al. 2019). A weak eosin solution was used at a rate of 1:99 before counting the cells, for evaluation of sperm concentration (×10^6^/ml) according to Smith and Mayer (1955) by the improved Neubauer haemocytometer slide (GmbH + Co., Brandstwiete 4, 2000 Hamburg 11, Germany). Percentages of live, dead, and abnormal spermatozoa were assessed using an eosin-nigrosin blue staining mixture (Blom 1950). The eosin stain penetrates cells with damaged membranes. Normal live spermatozoa do not take up the stain and appear white, while dead spermatozoa are stained pink. Healthy sperm typically have an oval head and a long tail, whereas abnormal spermatozoa may exhibit defects in the head, midpiece, or tail, such as a large or misshapen head or a crooked or double tail.

Eight semen samples were collected from each group at the end of the trial period. The samples were then centrifuged at 126× *g* for 20 min to separate the seminal plasma, which was stored at -20 °C for the determination of the total antioxidant capacity (TAC), reduced glutathione (GSH), malondialdehyde (MDA), and testosterone levels.

### Statistical analysis

In a completely randomized design, the differences among treatments were statistically analyzed with a one-way ANOVA test. The data was analyzed using SPSS (version 21, SPSS Inc., Chicago, IL, USA). The following mathematical model was used:

$$Yij = \mu + Ti + eij$$

Where *Yij*: Observations, *µ* = the overall mean, *Ti*: the fixed effect of treatment, *eij*: residual error. The significant differences among means were compared using Duncan’s new multiple-range test (Duncan 1955).

## Results

### Body temperature parameters

Impact of dietary supplements (SWBP_10_, SWBP_20_, BET_1_, or BET_2_) on parameters of body temperature (RT, ST, ET) and RR are presented in Table 2. During the overall trial period, all the tested feed additives have lowering effects (*P* < 0.05) on the apparent thermoregulation indices in the heat-stressed NZW rabbit bucks (HS-NZWB) compared to the control group. Among the tested supplements, the best (*P* < 0.001) values of RT, ET, and RR were recorded in SWBP groups, while the BET_2_ group showed the lowest value of ST (*P* = 0.004).

**Table 2:** Effect of dietary SWBP and BET on RT, ST, ET and RR in HS-NZWB

Items	CON	SWBP_10_	SWBP_20_	BET_1_	BET_2_	*P*-Value	Normal range
RT (˚C)	39.63 ^a^ ±0.03	39.14 ^c^ ±0.03	39.16 ^c^ ±0.04	39.31 ^b^ ±0.03	39.27 ^b^ ±0.03	< 0.001	38-38.6
ST (˚C)	39.53 ^a^ ±0.03	39.12 ^bc^ ±0.04	39.13 ^bc^ ±0.04	39.14 ^bc^ ±0.04	38.93 ^d^ ±0.26	0.004	37.8–38.3
ET (˚C)	37.53 ^a^ ±0.05	37.02 ^cd^ ±0.05	36.93 ^d^ ±0.05	37.10 ^bc^ ±0.06	37.1 9 ^b^ ±0.06	< 0.001	36-36.4
RR (breaths/minute)	104.93 ^a^ ±0.93	95.44 ^c^ ±0.82	94.02 ^c^ ±0.95	100.40 ^b^ ±0.92	98.32 ^b^ ±0.94	< 0.001	50–55

CON, SWBP_10_, SWBP_20_, BET_1_, and BET_2_: rabbit groups were fed the basal diets with no supplements, 10 and 20 g of willow bark powder (SWBP), or 1.0 and 2.0 g of betaine (BET) per kg of diet. RT: rectal temperature, ST: skin temperature, ET: ear temperature, and RR: respiration rate. a, b, c and d: means with different superscripts within the same rows are significantly different (*P* < 0.05)

### Serum biochemistry

Table 3 presents biochemical constituents in blood serum of the NZWB as impacted by the SWBP and BET supplementation. In comparison with the control, there were linear improvements in the concentrations of serum TL (*P* = 0.01) and TP (*P* < 0.001), as well as the activities of AST and ALT (*P* < 0.001) with all the tested supplements. Additionally, the ALB concentrations were significantly enhanced (*P* = 0.007) only with the high level of SWBP and BET. Also, the GLB levels were significantly upsurged in SWBP_20_, BET_1_, and BET_2_ groups. The serum TL concentrations were significantly lower in the SWBP groups (*P* = 0.01) compared to the BET and BET_2_ groups. The best values of serum proteins and lipids were recorded with the high level of BET, while the best values of ALT and AST were observed with the high level of SWBP.

**Table 3:** Effect of dietary SWBP and BET on serum biochemicals in HS-NZWB

Items		CON	SWBP_10_	SWBP_20_	BET_1_	BET_2_	*P-Value*
TP	g/dL	5.11^c^ ± 0.12	5.45^b^ ± 0.09	5.66^ab^ ± 0.06	5.54^b^ ± 0.05	5.83^a^ ± 0.10	< 0.001
ALB	g/dL	2.99^c^ ± 0.10	3.14^bc^ ± 0.04	3.22^ab^ ± 0.04	3.12^bc^ ± 0.04	3.35^a^ ± 0.04	0.007
GLB	g/dL	2.12^b^ ± 0.02	2.31^ab^ ± 0.08	2.44^a^ ± 0.08	2.42^a^ ± 0.08	2.48^a^ ± 0.07	0.010
ALB/GLB		1.42 ± 0.04	1.36 ± 0.05	1.32 ± 0.06	1.29 ± 0.06	1.35 ± 0.03	0.447
TL	mg/dL	427.87^a^ ± 2.94	397.99^b^ ± 6.56	372.08^b^ ± 5.25	340.91^c^ ± 6.3	334.41^c^ ± 4.28	0.010
AST	U/L	27.66^a^ ± 0.84	21.66^c^ ± 0.61	21.59^c^ ± 0.47	23.53 ^b^ ±0.4	22.48^bc^ ± 0.46	< 0.001
ALT	U/L	18.36^a^ ± 0.49	13.92^b^ ± 0.31	13.72^b^ ± 0.6	14.55 ^b^ ±0.42	14.52^b^ ± 0.32	< 0.001

CON, SWBP_10_, SWBP_20_, BET_1_, and BET_2_: rabbit groups were fed the basal diets with no supplements, 10 and 20 g of willow bark powder (SWBP), or 1.0 and 2.0 g of betaine (BET) per kg of diet. TP: total protein, ALB, Albumin, GLB: globulin, TL: total lipids, AST: aspartate amino transferase, and ALT: alanine amino transferase. a, b, and c: values with different superscripts within the same rows are significantly different (*P* < 0.05)

### Serum oxidative status

Incorporating SWBP or BET in the diets of Hs-NZWB resulted in significant improvements in all indices of serum oxidative status (*P* < 0.05) compared to the control (Table 4). There were no significant changes in GPx, GSH, and MDA values among all SWBP and BET groups. On the other side, among the tested doses of SWBP and BET, the high levels in the SWBP_20_ and BET_2_ rabbit groups triggered significant enhancements in SOD and TAC (*P* < 0.001) levels. The most useful impacts on oxidative status were observed with SWBP_20_ compared to the other tested groups.

**Table 4:** Effect of dietary SWBP and BET on serum oxidative status in in HS-NZWB

Items		CON	SWBP_10_	SWBP_20_	BET_1_	BET_2_	*P-Value*
SOD	ng/mL	23.11^d^ ± 0.16	24.98^c^ ± 0.59	28.70^a^ ± 0.69	25.47^bc^ ± 0.35	27.08^a^ ± 0.96	< 0.001
GP_X_	ng/mL	1.69^b^ ± 0.08	1.91^a^ ± 0.04	2.05^a^ ± 0.07	1.94^a^ ± 0.06	1.99^a^ ± 0.06	0.010
GSH	ng/mL	12.55^b^ ± 0.48	15.96^a^ ± 0.46	16.76^a^ ± 0.34	15.80^a^ ± 0.49	16.22^a^ ± 0.43	< 0.001
TAC	ng/mL	0.89^d^ ± 0.03	1.07^bc^ ± 0.06	1.24^a^ ± 0.03	1.03^c^ ± 0.05	1.19^a^ ± 0.02	< 0.001
MDA	nmol-mL	7.72^a^ ± 0.25	4.89^b^ ± 0.19	4.67^b^ ± 0.18	5.11^b^ ± 0.16	4.90^b^ ± 0.16	< 0.001

CON, SWBP_10_, SWBP_20_, BET_1_, and BET_2_: rabbit groups were fed the basal diets with no supplements, 10 and 20 g of willow bark powder (SWBP), or 1.0 and 2.0 g of betaine (BET) per kg of diet. TAC: total antioxidant capacity, GSH: reduced glutathione, GPx: glutathione peroxidase, SOD: superoxide dismutase and MDA: malondialdehyde. a, b, and c: values with different superscripts within the same rows are significantly different (*P* < 0.05)

### Testosterone levels in blood and seminal plasma, and reaction time

**Fig. 2:**
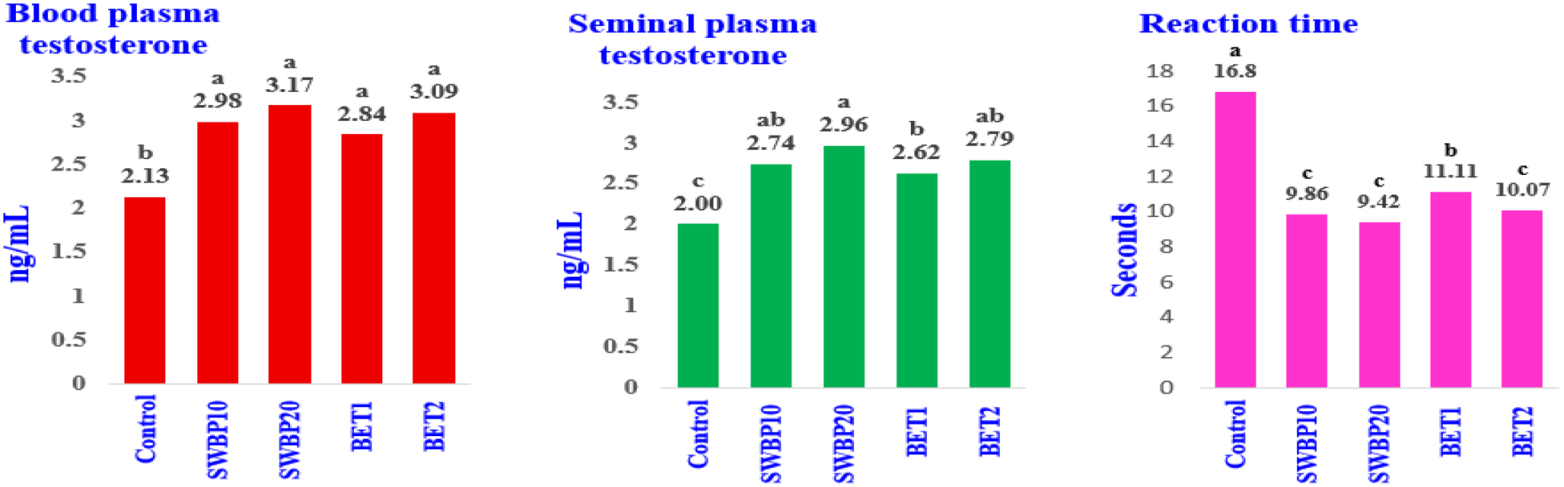
Effect of dietary SWBP and BET on testosterone levels in blood and seminal plasma, and reaction time of HS-NZWB. CON, SWBP _10_, SWBP _20_, BET_1_, and BET_2_: rabbit groups were fed the basal diets with no supplements, 10 and 20 g of willow bark powder (SWBP), or 1.0 and 2.0 g of betaine (BET) per kg of diet. a, b, and c: columns tagged with different letters represent means that differ significantly (*P* < 0.05)

Effects of supplementing the HS-NZWB with SWBP_10_, SWBP_20_, BET_1_, or BET_2_ on testosterone concentrations in blood and seminal plasma, and reaction time are shown in Fig. 2. Throughout the experimental period, all the tested additives considerably ameliorated the reaction time and levels of blood and seminal plasma testosterone compared to the control (*P* < 0.001). Among the examined supplements, the reaction time with the high level of SWBP (SWBP_20_) had the lowest value (*P* < 0.001), while there were no dose dependent impacts on reaction time and testosterone levels among the rest of the tested additives.

### Semen quality parameters

According to the results of Table 5, the dietary SWBP and BET significantly improved all the tested semen characteristics in the HS-NZWB (*P* < 0.05). The pH values of semen were similar for all supplementary doses of SWBP and BET, but they statistically (*P* = 0.001) lessened in parallel with that of the control. Only the high dose of SWBP and BET (SWBP _20_ and BET_2_) resulted in increased (*P* = 0.03) EV values compared to the control. Also, the SWBP_20_ group displayed a remarkable increase in SC (*P* < 0.001) among the tested groups. Moreover, the best values of EV, MM, PM, SC, LS, DS, and AS were detected in the SWBP_20_ group.

**Table 5:** Effect of dietary SWBP and BET on semen quality parameters in HS-NZWB

Items		CON	SWBP_10_	SWBP_20_	BET_1_	BET_2_	*P-Value*
pH		7.15 ^a^ ±0.01	7.07 ^b^ ±0.18	7.08 ^b^ ±0.012	7.07 ^b^ ±0.015	7.08 ^b^ ±0.01	0.001
EV	mL	0. 49 ^b^ ±0.03	0.54 ^ab^ ±0.03	0.61 ^a^ ±0.03	0.53 ^ab^ ±0.03	0.55 ^a^ ±0.02	0.030
MM		3.20 ^c^ ±0.14	4.40 ^a^ ±0.11	4.58 ^a^ ±0.10	3.94 ^b^ ±0.13	4.30 ^a^ ±0.11	< 0.001
PM	%	70.43 ^c^ ±1.46	83.28 ^a^ ±0.84	84.42 ^a^ ±1.06	77.89 ^b^ ±1.24	82.93 ^a^ ±0.79	< 0.001
SC	×10^6^	206.10 ^d^ ±5.14	291.47 ^b^ ±4.47	317.17 ^a^ ±4.72	264.50 ^c^ ±4.11	292.0 7 ^b^ ±3.34	< 0.001
LS	%	71.10 ^c^ ±0.89	82.92 ^a^ ±0.97	83.36 ^a^ ±0.96	78.23 ^b^ ±0.92	82.41 ^a^ ±0.66	< 0.001
DS	%	28.90 ^a^ ±0.89	17.08 ^c^ ±0.97	16.64 ^c^ ±0.96	21.77 ^b^ ±0.92	17.59 ^c^ ±0.66	< 0.001
AS	%	15.96 ^a^ ±0.43	11.19 ^c^ ±0.276	10.92 ^c^ ±0.31	12.11^b^ ± 0.31	11.48 ^bc^ ±0.23	< 0.001

CON, SWBP_10_, SWBP_20_, BET_1_, and BET_2_: rabbit groups were fed the basal diets with no supplements, 10 and 20 g of willow bark powder (SWBP), or 1.0 and 2.0 g of betaine (BET) per kg of diet. pH: concentration of hydrogen ions, EV: net ejaculate volume, MM: mass motility, PM: progressive motility, SC: sperm concentration, LS: live spermatozoa, DS: dead spermatozoa, and AS: abnormal spermatozoa. a, b, c, and d: values with different superscripts within the same rows are significantly different (*P* < 0.05)

### Oxidative biomarkers in seminal plasma

**Fig. 3:**
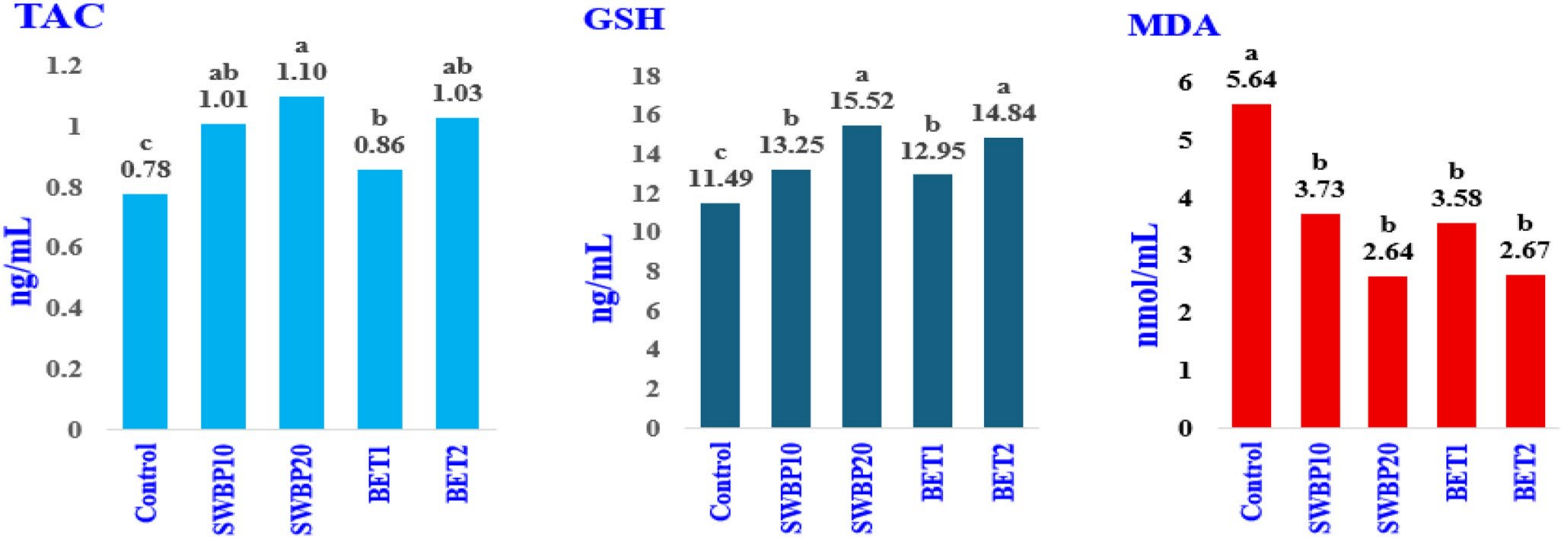
Effect of dietary SWBP and BET on total antioxidant capacity (TAC), reduced glutathione (GSH), and malondialdehyde (MDA) in seminal plasma of HS-NZWB. CON, SWBP_10_, SWBP_20_, BET_1_, and BET_2_: rabbit groups were fed the basal diets with no supplements, 10 and 20 g of willow bark powder (SWBP), or 1.0 and 2.0 g of betaine (BET) per kg of diet. a, b, and c: columns tagged with different letters represent means that differ significantly (*P* < 0.05)

The results of the tested levels of SWBP and BET on the oxidative biomarkers in the seminal plasma of HS-NZWB are presented in Fig. 3. It can be observed that the dietary SWBP and BET significantly increased levels of TAC (*P* = 0.004) and GSH (*P* < 0.001), while the MDA concentrations were significantly decreased as compared to the control (*P* < 0.001). Among the investigated additives, the GSH concentrations were ameliorated considerably (*P* < 0.001) with the high dose of SWBP and BET. The oxidative biomarkers were remarkable with the SWBP 20 group.

## Discussion

Animal performance is influenced by genetics, environment, and their interaction. HS is considered one of the environmental strains. It instigates significant health injury through OS, disrupted endocrine regulation, deteriorated immune function, and reproductive disorders. Eventually, this leads to reduced production performance and increased mortality rates in rabbits (Liang et al. 2022). Prolonged exposure of rabbits to HS can lead to physiological damage (Okab and Koriem 2008). This impairment is generated by OS, which results from the excessive production of ROS and/or a defect in the antioxidant system (Choi et al. 2009). Consequently, this can negatively impact the reproductive performance in rabbit bucks. The body temperature measurements in this study are consistent with the findings of El-Ratel et al. (2021), who reported that betaine acts as a body temperature modulator, reducing RT, ST, and ET in APRI bucks experiencing severe heat stress. Additionally, Chen et al. (2023) observed that RT, ST, and ET values tended to decrease with betaine supplementation (0.75 to 2 g/kg diet) in rabbits. Furthermore, Hassan et al. (2011) found that dietary BET supplementation (0.5 to 1 g/kg diet) linearly reduced RT in growing male NZW rabbits under heat stress conditions.

BET may help animals maintain a thermo-neutral state by reversing heat-induced disruptions of osmotic balance and preserving the tertiary structures of macromolecules in the kidneys (Huang et al. 2007). In simpler terms, the reduced body temperature is likely linked to osmoprotective mechanisms that assist in restoring cellular hydration, helping animals regulate their core body temperature (Ratriyanto and Mosenthin 2018; Deshpande et al. 2020). The abundant deposition of BET in cells or organelles can partially substitute inorganic ions to control osmotic pressure, thus relieving the damage caused by inorganic ions to enzymes and cell membranes (Lever and Slow 2010). This cytoprotective effect may be attributed to BET as a zwitterionic ion that can bind water and help stabilize the structure and function of proteins to regulate cell osmotic pressure (Kettunen et al. 2001).

The respiration rate of HS-NZWB showed a significant linear decrease (*P* < 0.001) in values (Table 2), consistent with findings from previous studies. Chen et al. (2023) found similar results when supplementing the diet of growing rabbits with BET (1 to 2 g/kg). Additionally, Hassan et al. (2011) and Daader et al. (2018) reported a reduction in respiration rate with dietary BET supplements in heat-stressed rabbits. Chen et al. (2023) concluded that BET can help maintain endocrine system homeostasis and effectively alleviate heat stress in rabbits.

This experiment demonstrated that SWBP lowered (*P* < 0.05) the body temperature indices and RR in the HS-NZWB. Similarly, the aqueous extract of *S. babylonica* leaves (100 mL/day) significantly reduced the panting rate, and mortality (*P* < 0.05) in the heat-stressed broiler chicks (Al-Fataftah and Abdelqader 2013). The bark of several species of willow is characterized by their antipyretic, analgesic, and anti-inflammatory properties (Mahdi et al. 2006). Willow trees are recognized for their ability to inhibit the actions of pyrogens, making them antipyretic agents. They reduce the activity of prostaglandin synthase with low selectivity and do not cause side effects (Khan 2017). The phenolic compounds in *Salix mucronata* leaves extract have a long history of usage as an analgesic, anti-inflammatory and anti-pyretic herbal remedy (Dissanayake et al. 2017). Likewise, Beltag et al. (2022) revealed that the anti-inflammatory properties of the *Salix* family may originate from their phytochemicals, including salicin, quercetin, and rutin. Safsaf willow bark has been reported to be rich in salicin (Aboul-Soud et al. 2020). The anti-inflammatory effects of salicin in *S. mucronata* are the inhibition of cyclooxygenase-1 (COX-1) and cyclooxygenase-2 (COX-2) leading to the inhibition of prostaglandin synthesis (Mahdi 2010; Beltag et al. 2022). In comparison with the activity of pure salicin, the salicin activity of the *S. mucronata* leaf extract may be enhanced by other metabolites involved in this extract (Dissanayake et al. 2017).

Evaluating the health and physiological status of rabbits can be achieved by analyzing their blood biochemistry. When augmenting a diet with any feed supplement, it is critical to monitor changes in serum metabolites to ensure the safety and efficacy of this supplement (Abdelghani et al. 2024). The serum profile indicates the climatic and management conditions to which the animals are exposed (Minafra et al. 2010).

BET supplements significantly improved the levels of serum proteins in the HS-NZWB (*P* < 0.05). The average values of TP, ALB, and GLB were upgraded by 14.09, 12.04, and 16.98%, respectively, in the BET_2_ group compared to the control group. These findings are matched Abu Hafsa et al. (2023) in NZW males and Hassan et al. (2012) in APRI bucks subjected to high ambient temperature. Likewise, Hassan et al. (2011) declared the beneficial impact of BET supplements on serum TP and GLB in NZW rabbits. Previous authors’ findings showed that BET supplementation advantageously influences dietary protein utilization and the intestinal integrity, which supports enhanced the levels of serum proteins. In the NZW rabbit males, BET significantly improved digestibility of the dietary protein (Abd El-Moniem et al. 2016; Abu Hafsa et al. 2023). As well, BET (0.75 to 2.0 g /kg basal diet) significantly increased TAC, GPx, and SOD activities, while significantly decreased MDA levels in the small intestine of the growing rabbits (Chen et al. 2023). Additionally, the dietary BET (1.25–2.5 g/ Kg diet) significantly augmented the activity of GPx in the gut, intestinal structure, activities of the digestive enzymes, and protein digestibility in piglets (Wang et al. 2020). The enhanced plasma ALB, GLB, and ALB/GLB ratio reveal the encouraging impact of BET as a methyl group donor on liver function by increasing protein synthesis (Hassan et al. 2005).

About ameliorating the activities of aminotransferases (*P* < 0.05) in the HS-NZWB with supplementing BET, the results are in concurrence with Hassan et al. (2012) who reported that BTE (1 g /Kg diet) reduced the activity of these enzymes in the serum of APRI rabbit bucks exposed to high ambient temperature. Moreover, previous studies on heat-stressed animals demonstrated that BET supplementation significantly (*P* < 0.05) reduced serum AST activity in NZW male rabbits (Hassan et al. 2011; at 1 g/Kg diet) and in Qixing rabbits (Li et al. 2024; at 1.5 g/Kg). BET is an osmolyte that stabilizes proteins, cell membranes, and cell organelles for animals under stressors (Attia et al. 2016; Ahmed et al. 2018). It possibly acts as hepatoprotective through their antioxidant activity by boosting the SOD and GP_X_ activities and GSH content and by upregulating the transcription of SOD1, GP_X_1, and uncoupling protein genes in the hepatocytes (Wen et al. 2021).

The lipid-lowering effect of BET in this scrutiny harmonized with findings of Abu Hafsa et al. (2023) in NZW rabbits and Attia et al. (2019) in roosters. Also, BET decreased the lipid content of the body in poultry (Metzler-Zebeli et al. 2009). Furthermore, BET has a sparing effect on methionine and choline (Hassan et al. 2005) and thus, increases choline for biosynthesis of very low-density lipoproteins, which prevent the lipid deposition via increased lipid removal from the liver (Yao and Vance 1989). On the other hand, the SWBP_20_ enhanced levels of serum TP, ALB, and GLB (*P* < 0.05). In rabbits, there was a tendency to elevating plasma TP and GLB levels with including the safaf willow (*Salix Safsaf*) twigs hay (7.5%) in diets of the NZW growing males (Basyony et al. 2018). However, the TP, ALB, and GLB contents in the chicks group received willow leaf extract (450 mg/kg diet) increased (*P* < 0.01) by 22.89, 63.33, and 19.63%, respectively, compared to the control group (Farag et al. 2024). Liver hepatocytes have an important role in the synthesis of most plasma proteins (Häussinger 1996). In liver tissues, significant (*P* = 0.001) linear advances of GSH and TAC pointed to that polyphenolic content in dietary powder of *S. alba* improved the oxidative status. This finding might be indicating that the bioactive compounds in the dietary willow bark have a hepatoprotective impact, thus it enhanced the liver functions (Panaite et al. 2020).

Concerning the TL concentrations in the HS-NZWB with SWBP addition, there were significant linear reductions in their values. Similar trend was noticed in TL of NZW growing male fed (7.5 to 22.5%) safsaf willow twigs hay (Basyony et al. 2018). Likewise, the broilers group fed graded levels of willow leaf extract showed a linear reduction (*P* < 0.01) in serum total cholesterol and triglycerides (Farag et al. 2024). Previous research stated that phenols could interact with cholesterol carriers and transporters present across the brush border membrane (Conseil et al. 1998; Leslie et al. 2001), thus resulting in lower cholesterol absorption (Zern and Fernandez 2005; Abdelnour et al. 2020).

Regarding results of serum ALT and AST, linear reductions (*P* < 0.05) were detected with SWBP addition. Similar results were detected by Basyony et al. (2018) in the NZW males. In the heat-stressed broilers, activities of serum ALT and AST were significantly lesser with 1% *Salix* bark extract diet (Saracila et al. 2018). Serum ALT and AST levels are sensitive indicators of hepatocellular damage (Zhang 2011; Martin et al. 2019). Phenolic compounds are included in the antioxidant defense of organism, protecting cellular damage from the detrimental impacts of ROS (Tan et al. 2018). Liver is a target of polyphenols. Under HS conditions, high levels of polyphenols which detected in the liver can elucidate their hepatoprotective effect (Hu et al. 2019). The orally administrated *S. mucronata* bark extract has a protective impact on liver cirrhosis in rats (Beltag et al. 2022). The in vitro phytochemical evaluation of *S. mucronata* exhibited that the TPC and total flavonoids content in SWPB were 126.33 mg GAE and 79.01 mg QE per gram dry weight of SWPB, respectively.

In animals, prior research papers exposed the beneficial impacts of phenolic compounds detected in many plants. The anti-lipogenic effect of SWBP can be attributed to gallic acid (Olaniyan et al. 2018), quercetin (Jahan et al. 2018), and rutin (Al-Rejaie et al. 2013). Also, the reduction in liver transaminases may be due to the rutin activity (Khan et al. 2012) and caffeic acid (Liu et al. 2021). These compounds were detected in *S. safsaf*, *S. alba*, and *S. mucronata* as published by Aboul-Soud et al. (2020), Kalia et al. (2021), and Ahmad et al. (2023), respectively.

During the hot Egyptian summer, the THI were elevated, accordingly, NZWB are prone to OS. The ROS are released excessively into the blood, resulting in an imbalanced redox status (Jimoh et al. 2017). ROS must be safely scavenged through administering antioxidant compounds to avoid the detrimental impacts of the OS (Oriani et al. 2001; El-Ratel et al. 2025). The OS biomarkers (SOD, GPx, GSH, TAC, and MDA) in blood can reveal the oxidative status of animals. In the current study, serum levels of GPx, SOD, GSH, MDA, and TAC in HS-NZWB were notably better with SWBP or BET supplements.

In farm animals, BET, a is a novel compound and has been found to ameliorate HS in rabbits (Hassan et al. 2011). Values of SOD, GPx, GSH, TAC, and MDA were improved by 17.18, 17.75, 29.24, 33.71, and 36.52%, respectively, in BET2 group relative to the control. These findings are consentient with Abu Hafsa et al. (2023) in the NZW male rabbits and Zhang et al. (2014) and Lakhani et al. (2019) in cattle under HS conditions. Those authors found that BET supplements improved the oxidative biomarkers in blood. BET has various mechanisms to reduce the negative effects of HS in animals. It plays vital functions in combating redox imbalance during the HS (Uyanga et al. 2022). The valuable capabilities of BET could be through protecting the mitochondrial function (Khodayar et al. 2018), modifying cysteine supply in the transsulfuration pathway for GSH synthesis, enhancing methionine and S-adenosylmethionine, which form a protective membrane around cells by regulating the cycle of methionine-homocysteine (Zhang et al. 2016), modulating the endogenous antioxidant enzymes and GPx mRNA expression (Cai et al. 2021). On the other hand, the high dietary SWBP level (SWBP_20_) enhanced the percentages of SOD, GPx, GSH, TAC, and MDA were 24.19, 21.30, 33.55, 39.33, and 39.51%, respectively, relative to the control. Similar results were reported by previous authors. Zabihi et al. (2018) recorded that the polyphenolic compounds in *S. alba* twigs’ extract inhibited the OS through their potency of free-radical scavenging and ameliorating the levels of SOD, GSH, and MDA in the hypercholesterolaemic rabbits. Ishikado et al. (2013) reported that the polyphenolic compounds in willow bark extract combated the OS through activation of the transcription factor Nrf2 which improves the intracellular GSH in human umbilical vein endothelial cells.

Salicin, phenolic acids (gallic, chlorogenic, syringic, *p*-coumaric and *trans*-cinnamic acids) and flavonoids were identified in the bark of various *Salix* species (Gligorić et al. 2023). The phenolic compounds in the extract of *S. mucronata* leaves showed strong antioxidant activity in vitro due to their ability to inhibit lipid peroxidation (LPO) (Dissanayake et al. 2017). Gallic acid prevents cell damage through impeding the unruly generation of ROS. The antioxidant activity of gallic acid is related to its efficient electron transfer capacity (Velderrain-Rodríguez et al. 2018). Chlorogenic acid has demonstrated powerful biological activities, particularly its role as an antioxidant (Mussatto et al. 2011). In vitro, chlorogenic acid can scavenge free radicals and impede the OS (Mohammadi and Hosseinchi Gharehagha 2024). The *ρ*-Coumaric acid has an antioxidant activity through quenching the radicals that responsible for LPO in goats (Akhtarshenas et al. 2024). The adequate levels of coumaric acid and cinnamic acid can alleviate the HS. The antioxidant activity of rutin occurs by directly removing ROS through the donation of electrons to free radicals (Ghiasi et al. 2012). The dietary quercetin improves the plasma antioxidant levels and reduces the ROS (Hu et al. 2015). Quercetin lowered the serum MDA concentration in NZW adult male rabbits by scavenging the oxyradicals from the lipid bilayer of the cell (Naseer et al. 2020).

The detrimental effect of HS on Leydig cells was proved in reducing testosterone production and libido in the heat-stressed rabbit bucks (Hosny et al. 2020; Yousef et al. 2022). The natural antioxidant supplements augmented the testosterone synthesis and subsequently the libido in the heat-stressed APRI rabbits (El-Ratel et al. 2021). In the present study, under HS conditions, the SWBP and BET significantly improved testosterone levels in both serum and seminal plasma. Thus, the sexual desire was advanced in the supplemented groups (*P* < 0.001).

In a dose-dependent manner, the reaction time was linearly shrunk with adding BET (*P* **<** 0.001). The reaction time was reduced by 40.06% in BET_2_ relative to the control. These results are in correspondence with those attained by El-Ratel et al. (2021) in rabbit bucks suffering from HS. They found that dietary BET (1 g/Kg diet) significantly increased plasma testosterone (*P* < 0.012), while significantly decreased the reaction time (*P* < 0.001), compared with the control. The improvement in sexual desire of rabbit bucks under HS may be attributed to the thermoregulatory action of BET (Table 2), which is attributed to its abilities as a natural anti-heat stress mediator for farm animals (Abu Hafsa et al. 2023), a methyl group donor (Cronje 2018), and an antioxidant and anti-inflammatory (Zhao et al. 2018).

Likewise, the reaction time was lowered with SWBP supplements. It decreased by 41.31 and 43.93% in SWBP_10_ and SWBP_20_, respectively, compared to the control. Our data from the in vitro phytochemical assay detected considerable levels of TPC (126.33 mg GAE) and TFC (79.01 mg QE) in each gram of SWBP. Dissanayake et al. (2017) stated that the phenolic compounds in *S. mucronata* have antipyretic anti-inflammatory properties. Saracila et al. (2021) added that the natural antioxidants in willow bark are responsible for their useful impact. Supplementing the aged roosters with gallic acid considerably elevated the plasma testosterone levels (*P* < 0.01), indicating protective activities of the steroidogenic pathways in Leydig cells (Ghadimi et al. 2024). In Wistar rats exposed to scrotal hyperthermia, the dietary rutin mitigating the oxidative stress by boosting the activities of the antioxidant enzymes and reducing LPO (Olasehinde et al. 2025). Several studies indicated declined semen characteristics and damage to sperm DNA, which is due to OS in heat-stressed mammals (Hosny et al. 2020; Ijab et al. 2022). In this research, the qualitative and quantitative enhancements in the measured semen parameters (EV, MM, and PM, SC, LS, and sperm normality) compared to the control group were attributed to the SWBP and BET as natural exogenous antioxidants.

BET, particularly the high level (BET_2_), yielded notable improvements in all the tested semen characteristics of the HS-NZWB (*P* < 0.05). In the current experiment, findings of semen quality are in the same line with El-Ratel et al. (2021), who reported that BET supplements upgraded the EV, PM, SC, viability, normality, intact acrosome, and membrane integrity in the heat-stressed APRI rabbit bucks (*P* < 0.05). Likewise, Hassan et al. (2012) found that the dietary BET (1 g/Kg diet) significantly improved the EV, SC, sperm motility, and viability (*P* < 0.05) in rabbits under HS. From the parameters discussed above, the roles of BET, particularly with the high dose (BET_2_), as an antipyretic and antioxidant, can explain the improvement in semen quality through restoring serum testosterone concentration and supporting spermatogenesis. The noticeable decrease in acrosomal damage and sperm abnormality in APRI bucks is dependent on enhancing the testosterone level (El-Ratel et al. 2023). Also, BET increased the testicular protection against the ROS and enhanced the germinal epithelium activity in mice subjected to HS (Shadmehr et al. 2018). Additionally, semen characteristics are negatively impacted by the homocysteine produced during HS. The dietary BET decreased the homocysteine concentrations. BET is a methyl donor that contributes to the conversion of homocysteine into methionine. This reaction is mediated by betaine-homocysteine methyl transferase (Rokade et al. 2020). Thus, BET has a methionine-sparing effect, which enhances GSH synthesis and protects the cell from ROS (Zhang et al. 2016). SWBP_20_ exhibited the best semen characteristics in the HS-NZWB (*P* < 0.05), where the pH, EV, MM, PM, SC, LS, and AS values were improved by 0.98, 24.49, 43.13, 19.86, 53.89, 17.24, and 31.58%, respectively, relative to the control.

Chemical composition varies among willow species and plant organs of the same species (Gligorić et al. 2023). Previous studies have shown high concentrations of phenolic compounds and flavonoids in *S. mucronata* leaves (Dissanayake et al. 2017; Ahmad et al. 2023). In this study, sun-dried S. mucronata bark was found to have significant TPC and TFC (126.33 mg GAE and 79.01 mg QE/g, respectively). Polyphenols, known for their antioxidant properties, can help alleviate heat stress by donating hydrogen atoms to free radicals and quenching ROS (Hu et al. 2019). The bark of Salix species contains phenolic acids (gallic, chlorogenic, syringic, p-coumaric, and trans-cinnamic acids) and flavonoids (epicatechin, rutin, quercetin, and naringenin), which have antioxidant and anti-inflammatory effects (Kalia et al. 2021). *S. alba* leaf extract has been used as a phytogenic feed additive to improve animal performance during heat stress. However, the efficacy of S. mucronata bark has not been compared in terms of semen quality in rabbits.

In this study, the antipyretic and antioxidant properties of SWBP, especially the high dose (SWBP_20_) may elucidate the improvement in HS-NZWB semen quality. Sertoli cells play a crucial role in the development of germ cells into spermatozoa. The regulation of spermatogenesis is influenced by the actions of follicle-stimulating hormone (FSH) and testosterone on Sertoli cells. While testosterone is essential for spermatogenesis, FSH primarily enhances spermatogenic output by increasing the number of Sertoli cells (Griswold 1998). For Sertoli cells to function properly in the testis, testosterone signaling is necessary, which operates through both classical and non-classical pathways (Walker 2009).

Gallic acid supplements increased testosterone levels without affecting basal LH or FSH levels, indicating direct protective actions on Leydig cell steroidogenic pathways instead of modifying pituitary gonadotropin set points. Additionally, the antioxidant potency of gallic acid boosted the integrity of sperm plasma membrane, SC, and sperm morphology by alleviating testicular oxidative damage (Ghadimi et al. 2024). In the HS-NZWB, dietary quercetin enhanced semen quality (Naseer et al. 2018). Moreover, the quercetin supplements maintained the seminiferous tubules, Leydig cells, germinal and Sertoli cells in the HS-NZWB by reducing the OS. These protective effects led to significant (*P* < 0.05) improvements in PM, SC, and viability (Naseer et al. 2020). The forementioned HS relief and antioxidant properties of SWBP may explain the improvement that occurred in semen quality of HS-NZWB in this study.

The pH values of semen were found to be statistically lower (*P* = 0.001) compared to the control group by approximately 1%. Theau-Clément et al. (2016) reported a significant negative correlation between fertility and semen pH value. They observed that even a slight increase in semen pH led to a decrease in fertility rates in rabbits.

Lipids are major structural and functional components of sperm, and their composition can change due to physiological events (Mourvaki et al. 2010). Sperm contains a high concentration of polyunsaturated fatty acids, which makes it vulnerable to LPO, especially due to the significant production of ROS (Hezavehei et al. 2018). HS leads to an elevation in LPO, hence negatively affects sperm quality (Attia et al. 2019). To combat this OS, sperm employs various intrinsic antioxidant protective systems found both in the sperm itself and in the seminal plasma. These systems include both enzymatic and non-enzymatic antioxidants (Amidi et al. 2016). The overproduction of free radicals in seminal plasma damages sperm membranes and mitochondria (Mizera et al. 2019).

In the HS-NZWB seminal plasma, natural exogenous antioxidants (BET and SWBP) significantly improved oxidative biomarkers. MDA, GSH, and TAC values increased by 52.65%, 29.15%, and 32.05%, respectively, in the BET2 group compared to the control. El-Ratel et al. (2021) also observed similar improvements in APRI rabbit bucks with dietary BET supplementation. They found that BET enhanced the chemical composition of seminal plasma, leading to positive effects on spermatozoa function and metabolism. MDA levels decreased by 37.84%, while GSH and TAC increased by 59.62% and 30.95%, respectively, in the seminal plasma compared to the control. BET’s lipotropic activity allows it to easily cross cell membranes and protect against oxidative stress. Furthermore, the beneficial effects of BET may be linked to its role as ROS scavenger in testicular tissues, reducing endogenous oxidative damage (El-Speiy et al. 2017). The SWBP_20_ group improved MDA, GSH, and TAC levels in seminal plasma by 53.19%, 35.07%, and 41.02%, respectively, compared to the control. In the same line, willow bark extract can reduce the OS by improving GSH concentrations in animals (Sharma et al. 2011; Nauman et al. 2018) and humans (Ishikado et al. 2013).

In this respect, phenolics can serve as antioxidants and have the potential to protect sperm from OS (Seddiki et al. 2017). In this study, the in vitro phytochemical evaluation of *S. mucronata* willow bark exhibited high TPC and TFC. The pentahydroxyflavone structure of quercetin functions as a chelating agent for metal ions due to its ortho-dihydroxy phenolic structure, and it scavenges lipid alkoxyl and peroxyl radicals (Naseer et al. 2020). Gallic acid showed potent free radical scavenging abilities by improving SOD activity, TAC, and MDA values in the aged rooster seminal plasma, thereby preserving sperm plasma membrane, followed by the maintenance of semen quality traits (Ghadimi et al. 2024). It has inhibitory effects against the ROS generation and LPO (Jahan et al. 2018). The dietary flavonoid rutin alleviated OS in male by boosting the activities of antioxidant enzymes, decreasing LPO, and suppressing inflammatory (Olasehinde et al. 2025).

Despite the promising results of this study, we have some limitations including exploring the molecular pathways to enhance our understanding of how SWBP or BET improve reproductive health. Furthermore, there is a limitation regarding the histological studies regarding testicular morphometrics and exploring the immune expression of some related biomarkers to provide more insights on the anti-heat stress effects of both additions. Additionally, using omics tools to explore in-depth the different doses of SWBP on the HS-bucks in natural environments should also be provided.

## Conclusions

The dietary supplements SWBP or BET improved all the tested parameters in the HS-NZWB. Among the tested supplements, BET at a dosage of 2 g/Kg diet (BET_2_) yielded the best results in terms of ST and levels of serum proteins and TL. However, the other apparent physiological indices, along with serum ALT and AST activities, serum and seminal testosterone levels, and oxidative biomarkers, sexual desire, and semen quality showed superior values with SWBP at 20 g/Kg diet (SWBP_20_) compared to the un-supplemented HS-NZWB. Hence, it is recommended to use SWBP_20_ and/or BET_2_ as an effective strategy for enhancing the reproductive efficiency of the breeding rabbit bucks under HS conditions.

## Data Availability

All data generated or analyzed during this study are included in this published article.

## References

[CR1] Abd El-Ghany WA, Babazadeh D (2022) Betaine: A potential nutritional metabolite in the poultry industry. Animals 12:2624. 10.3390/ani12192624 36230366 10.3390/ani12192624 PMC9559486

[CR2] Abd El-Moniem A, Elham A, Daader AH, Al-Sagheer AA, Gabr HA (2016) Effect of vitamin C, vitamin E or betaine addition on alleviation of heat stress impacts on growing rabbits. Zagazig J Agric Res 43(5):1601–1613

[CR4] Abdel-Khalek AM (2013) Supplemental antioxidants in rabbit nutrition: a review. Livest Sci 158:95–105

[CR3] Abdelghani IG, Sheiha AM, Abdelnour SA, El-Maati MFA, El-Darawany AA, Al-Marakby KM (2024) Dietary supplement guava leaf extract regulates growth, feed utilization, immune function, nutrient digestibility and redox regulation in growing rabbits. Trop Anim Health Prod 56(8):325 39361143 10.1007/s11250-024-04126-4 PMC11450086

[CR6] Abdelnour SA, El-Saadony MT, Saghir SAM, Abd El-Hack ME, Al-Shargi OYA, Al-Gabri N, Salama A (2020) Mitigating negative impacts of heat stress in growing rabbits via dietary prodigiosin supplementation. Livest Sci 240:104220

[CR5] Abdelnour SA, Al-Gabri NA, Hashem NM, Gonzalez-Bulnes A (2021) Supplementation with proline improves haemato-biochemical and reproductive indicators in male rabbits affected by environmental heat-stress. Animals 11(2):373 33540779 10.3390/ani11020373 PMC7913087

[CR7] Abo-Elsoud MA, Hashem NM, Nour El-Din ANM, Kamel KI, Hassan GA (2019) Soybean isoflavone affects in rabbits: effects on metabolism, antioxidant capacity, hormonal balance and reproductive performance. Anim Reprod Sci 203 10.1016/j.anireprosci.2019.02.007 30819569

[CR8] Aboul-Soud MAM, Ashour AE, Challis JK, Ahmed AF, Kumar A, Nassrallah A, Alahmari TA, Saquib Q, Siddiqui MA, Al-Sheikh Y (2020) Biochemical and molecular investigation of in vitro antioxidant and anticancer activity spectrum of crude extracts of willow leaves. Plants vol. 9, issue 10 10.3390/plants9101295 PMC7599573 33008079

[CR9] Abu Hafsa SH, Centoducati G, Hassan AA, Maggiolino A, Elghandour MMMY, Ahmad GM, Abu Serie MM, Abdel-Latif MS, Ghoneem T, Ghareeb DA, Yacout GA (2023) Potential anti-proliferative activity of Salix mucronata and triticum Spelta plant extracts on liver and colorectal cancer cell lines. Sci Rep 13(1):3815 36882428 10.1038/s41598-023-30845-z PMC9992471

[CR127] Ahmad GM, Abu Serie MM, Abdel-Latif MS, Ghoneem T, Ghareeb DA, Yacout GA (2023) Potential anti-proliferative activity of Salix mucronata and Triticum spelta plant extracts on liver and colorectal cancer cell lines. Sci Rep 13:3815. 10.1038/s41598-023-30845-z 10.1038/s41598-023-30845-z PMC9992471 36882428

[CR10] Ahmed Mervat MN, Ismail Z, Abdel-Wareth A (2018) Application of betaine as feed additives in poultry nutrition – a review. J Exp Appl Anim Sci 2:266–272. 10.20454/jeaas.2018.1428

[CR11] Akhtarshenas B, Kowsar R, Hajian M, Vash NT, Soltani L, Mahdavi AH, Esfahani MHN (2024) ρ-Coumaric acid-zinc oxide nanoparticles improve post-thaw quality of goat spermatozoa and developmental competence of fertilized oocytes in vitro. Sci Rep 14(1):31971 39738447 10.1038/s41598-024-83585-z PMC11686304

[CR14] Al-Rejaie SS, Aleisa AM, Sayed-Ahmed MM, Al-Shabanah OA, Abuohashish HM, Ahmed MM, Hafez MM (2013) Protective effect of Rutin on the antioxidant genes expression in hypercholestrolemic male Westar rat. BMC Complement Altern Med 13:1–9 23773725 10.1186/1472-6882-13-136 PMC3717094

[CR13] Alipour M, Mohammadi M, Zarghami N, Ahmadiasl N (2006) Influence of chronic exercise on red cell antioxidant defense, plasma malondialdehyde and total antioxidant capacity in hypercholesterolemic rabbits. J Sci Med Sport 5:682 PMC3861771 24357965

[CR15] Amidi F, Pazhohan A, Shabani Nashtaei M, Khodarahmian M, Nekoonam S (2016) The role of antioxidants in sperm freezing: a review. Cell Tissue Bank 17:745–756 27342905 10.1007/s10561-016-9566-5

[CR16] Anderson ME (1985) Determination of glutathione and glutathione disulfide in biological samples. Methods Enzymol 113:548–555 4088074 10.1016/s0076-6879(85)13073-9

[CR17] AOAC (2000) Association of Official Analyical Chemists Official Methods of Analysis 1 7th Ed. Published the AOAC. Washington DC

[CR18] Arumugam MK, Paal MC, Donohue TM, Ganesan M, Osna NA, Kharbanda KK (2021) Beneficial effects of betaine: a comprehensive review. Bio 10(6):456 10.3390/biology10060456 PMC8224793 34067313

[CR19] Attia YA, Abd El­Hamid AEE, Abedalla AA (2016) Laying performance, digestibility and plasma hormones in laying hens exposed to chronic heat stress as affcted by betaine, vitamin C, and/or vitamin E supplementation. Springerplus 5:1619 27652192 10.1186/s40064-016-3304-0 PMC5028346

[CR20] Attia YA, El­Naggar AS, Abou­Shehema BM, Abdella AA (2019) Effect of supplementation with trimethylglycine (betaine) and/or vitamins on semen quality, fertility, antioxidant status, DNA repair and welfare of roosters exposed to chronic heat stress. Animals 9:547 31408981 10.3390/ani9080547 PMC6719041

[CR21] Bai R, Guo J, Ye XY, Xie Y, Xie T (2022) Oxidative stress: the core pathogenesis and mechanism of alzheimer’s disease. Ageing Res Rev 77:101619 35395415 10.1016/j.arr.2022.101619

[CR22] Basyony MM, Eman I, Abd El Gawad D, Dohreig RMA (2018) Evaluation of Egyptian tree Willow (Salix Safsaf) leaf and its feeding Af fect on pro-ductive performance of new Zealand white rabbits. Egypt J Rabbit Sci 28(1):39–62

[CR23] Beltag DM, Ali EMM, Sakr HMS, Radwan EH (2022) Biochemical studies on the effect of prostaglandin inhibitors and Willow bark extract on liver cirrhosis induced by acetylsalicylate in rats. J Med Life Sci 4(4):82–104

[CR24] Blom E (1950) A one-minute live–dead sperm stain by meansof eosin–nigrosin. Fertil Steril 1:176–177

[CR25] Cai Y, Deng M, Zhang Q, Liu Z, Wang L, Sheng W, Zhang Y, You P, Wang Z, Wang F (2021) Effects of dietary betaine supplementation on biochemical parameters of blood and testicular oxidative stress in Hu sheep. Theriogenology Apr 1:164:65–73. 10.1016/j.theriogenology.2021.01.006 10.1016/j.theriogenology.2021.01.006 33556906

[CR26] Chen X, Li Z, Pu J, Cai J, Zhao H, Jia G, Liu G, Tian G (2023) Dietary betaine improves the intestinal health and growth performance of heat-stressed growing rabbits in summer. J Anim Sci Jan 3:101 10.1093/jas/skad363 PMC10684048 37875147

[CR27] Choi SI, Kim TI, Kim KS, Kim BY, Ahn SY, Cho HJ, Lee HK, Cho HS, Kim EK (2009) Decreased catalase expression and increased susceptibility to oxidative stress in primary cultured corneal fibroblasts from patients with granular corneal dystrophy type II. Am J Pathol 175:248–261 19497990 10.2353/ajpath.2009.081001 PMC2708811

[CR28] Conseil G, Baubichon-Cortay H, Dayan G, Jault JM, Barron D, Di Pietro A (1998) Flavonoids: a class of modulators with bifunctional interactions at vicinal ATP-and steroid-binding sites on mouse P-glycoprotein. Proc Natl Acad Sci 95(17):9831–9836 9707561 10.1073/pnas.95.17.9831 PMC21422

[CR29] Cronje PB (2018) Essential role of Methyl donors in animal productivity. Anim Prod Sci 58(4):655–665

[CR31] Daader AH, Yousef MK, Abdel-Samee AM, Abd El-Nour SA (2016) Recent trends in rabbit reproductive management: special reference to hot regions. 11 th World Rabbit conference. Qingdao china p 149–166

[CR30] Daader AH, Al­Sagheer AA, Gabr HA, Abd El­Moniem EA (2018) Alleviation of heat­stress­related physiological perturbations in growing rabbits using natural antioxidants. Span J Agric Res 16:e0610

[CR32] De Blas C, Mateos GG (2010) Feed formulation. In: de Blas C, Wiseman J (eds) Nutrition of the rabbit – 2nd edition. CAB International, UK

[CR33] Deshpande A, Singh SV, Somagond YM, Sheoran P, Naskar S, Chahal VP (2020) Physio-biochemical responses and growth performance of Buffalo heifers to betaine supplementation during hot humid season under field conditions. Indian J Anim Sci 90(3):416–423

[CR34] Dissanayake AA, Zhang CR, Gaber MKA, Nair MG (2017) Salicylic glycosides in Salix mucronata with antioxidant and anti-inflammatory activities. Nat Prod Commun 12(11)

[CR35] Duncan DB (1955) Multiple range and multiple F tests. Biomet 11(1):1–42

[CR36] El-Ratel IT, Attia KAH, El-Raghi AA, Fouda SF (2021) Relief of the negative effects of heat stress on semen quality, reproductive efficiency and oxidative capacity of rabbit bucks using different natural antioxidants. Anim Biosci 34:844–854 32819074 10.5713/ajas.20.0258 PMC8100465

[CR37] El-Ratel IT, Elbasuny ME, El-Nagar HA, Abdel-Khalek AE, El-Raghi AA, El Basuini MF, El-Kholy KH, Fouda SF (2023) The synergistic impact of spirulina and selenium nanoparticles mitigates the adverse effects of heat stress on the physiology of rabbit’s bucks. PLoS One Jul 12(7):e0287644 10.1371/journal.pone.0287644 PMC10337889 37437098

[CR38] El-Ratel IT, Mekawy A, Hassab SH, Abdelnour S (2025) Enhancing growing rabbit heat stress resilience through dietary supplementation with natural antioxidants. BMC Vet Res 21(1):28 39827123 10.1186/s12917-024-04466-1 PMC11748597

[CR39] El-Speiy ME, Khaled FA, ElHanoun AM (2017) Effect of ginger supplementation on reproductive performance of male rabbits. Glob Sci J Biol 2:2631

[CR40] Farag SA, El-Keredy A, Abd El Gawad SA, Swelum AA, Tellez-Isaias G, Abouzeid AE (2024) Impacts of Willow (Salix Babylonica L.) leaf extract on growth, cecal microbial population, and blood biochemical parameters of broilers. Poult Sci 103(3):103386 38176372 10.1016/j.psj.2023.103386 PMC10805942

[CR12] Fataftah A, Abdelqader A-R, Anas (2013) Improving performance of laying hens in hot regions by desert coolers. Int J Poult Sci 12:590–595

[CR41] Fekete S, Gippert T (1986) Digestibility and nutritive value of nineteen important feedstuffs for rabbits. J Appl Rabbit Res 9:103–108

[CR42] Flohé L, Günzler WA (1984) Assays of glutathione peroxidase. Methods Enzymol 105:114–121 6727659 10.1016/s0076-6879(84)05015-1

[CR48] Gajardo-Parra NF, Meneses L, Duarte ARC, Paiva A, Held C (2022) Assessing the influence of betaine-based natural deep eutectic systems on horseradish peroxidase. ACS Sustain Chem Eng 10:12873–12881. 10.1021/acssuschemeng.2c04045 36573121 10.1021/acssuschemeng.2c04045 PMC9783073

[CR43] Ghadimi M, Sharifi SD, Najafi A, Mohammadi H (2024) Gallic acid supplementation partially ameliorates reproductive aging in rooster breeders by improving semen quality, sperm kinetics, hormones, and antioxidant status. Poult Sci Jul 103(7):103842 10.1016/j.psj.2024.103842 PMC11154696 38806003

[CR44] Ghiasi M, Azadnia A, Arabieh M, Zahedi M (2012) Protective effect of Rutin (vitamin p) against Heme oxidation: A quantum mechanical approach. Comput Theor Chem 996:28–36

[CR45] Ghiselli A, Serafini M, Natella F, Scaccini C (2000) Total antioxidant capacity as a tool to assess redox status: critical view and experimental data. Free Radic Biol Med 29:1106–1114 11121717 10.1016/s0891-5849(00)00394-4

[CR46] Gligorić E, Igić R, Teofilović B, Grujić-Letić N (2023) Phytochemical screening of ultrasonic extracts of Salix species and molecular Docking study of Salix-derived bioactive compounds targeting pro-inflammatory cytokines. Int J Mol Sci 24(14):11848 37511606 10.3390/ijms241411848 PMC10380267

[CR49] Gouda NH, El-Kelawy HM, Abd-El-Rahim M, El-Haded RA, Abdelnour SA, Elmansy S, Elkashef AA, Abd-Allah MS (2025) Dietary betaine for fattening Californian rabbits: in vivo and in silico insights into growth, blood physiology, and nutrient utilization. Trop Anim Health Prod Jul 21;57(7):312. 10.1007/s11250-025-04554-w 10.1007/s11250-025-04554-w 40691331

[CR47] Griswold MD (1998) The central role of Sertoli cells in spermatogenesis. Semin Cell Dev Biol 9(4):411–416. 10.1006/scdb.1998.0203 9813187 10.1006/scdb.1998.0203

[CR52] Hassan RA, Attia YA, El–Ganzory EH (2005) Growth, carcass quality, and blood serum constituents of slow growth chicks as affected by betaine additions to diets containing 1. Different levels of choline. Int J Poult Sci 4:840–850

[CR51] Hassan RA, Ebeid TA, Abd El-Lateif AI, Ismail NB (2011) Effect of dietary betaine supplementation on growth, carcass and immunity of new Zealand white rabbits under high ambient temperature. Liv Sci 135:103–109

[CR50] Hassan RA, Morsy WA, Abd El-Lateif AI (2012) Effect of dietary ascorbic acid and betaine supplementation on semen characteristics of rabbit bucks under high ambient temperature. Proceedings of the 10th World Rabbit Congress (WRSA), Sharm El-Sheikh, Egypt. 273–277

[CR53] Häussinger D (1996) Physiological functions of the liver. In Comprehensive Human Physiology: From Cellular Mechanisms to Integration pp. 1369–1391

[CR54] Hezavehei M, Sharafi M, Kouchesfahani HM, Henkel R, Agarwal A, Esmaeili V, Shahverdi A (2018) Sperm cryopreservation: A review on current molecular cryobiology and advanced approaches. Reprod Biomed Online 37(3):327–339 30143329 10.1016/j.rbmo.2018.05.012

[CR55] Hosny NS, Hashem NM, Morsy AS, Abo-Elezz ZR (2020) Effects of organic selenium on the physiological Response, blood Metabolites, redox Status, semen Quality, and fertility of rabbit bucks kept under natural heat stress conditions. Front Vet Sci Jun 12:7:290 10.3389/fvets.2020.00290 PMC7303341 32596265

[CR56] Hu J, Yu Q, Zhao F, Ji J, Jiang Z, Chen X, Gao P, Ren Y, Shao S, Zhang L, Yan M (2015) Protection of Quercetin against triptolide-induced apoptosis by suppressing oxidative stress in rat Leydig cells. Chem Biol Interact 240:38–46 26277538 10.1016/j.cbi.2015.08.004

[CR57] Hu R, He Y, Arowolo MA, Wu S, He J (2019) Polyphenols as potential attenuators of heat stress in poultry production. Antioxidants 8(3):67 30889815 10.3390/antiox8030067 PMC6466569

[CR58] Huang QC, Xu ZR, Han XY, Li WF (2007) Effect of betaine on growth hormone pulsatile secretion and serum metabolites in fiishing pigs. J Anim Physiol Anim Nutr 91:85–90 10.1111/j.1439-0396.2006.00644.x 17355337

[CR59] Ijab R, Ayen E, Khaki A, Soleimanzadeh A (2022) Evaluation of dietary betaine on post-thawed semen quality in mature bulls during summer heat stress. J Ovarian Res 13(1):61 10.30466/vrf.2020.124845.2927 PMC9094597 35601790

[CR60] IRRG (2005) International rabbit reproduction Group. Guidelines for the handling of rabbit bucks and semen. World Rab Sci 13:71–91

[CR61] Ishikado A, Sono Y, Matsumoto M, Robida-Stubbs S, Okuno A, Goto M, Makino T (2013) Willow bark extract increases antioxidant enzymes and reduces oxidative stress through activation of Nrf2 in vascular endothelial cells and caenorhabditis elegans. Free Radic Biol Med 65:1506–1515 23277146 10.1016/j.freeradbiomed.2012.12.006 PMC3800243

[CR62] Jahan S, Abid A, Khalid S, Afsar T, Shaheen G, Almajwal A, Razak S (2018) Therapeutic potentials of Quercetin in management of polycystic ovarian syndrome using letrozole induced rat model: a histological and a biochemical study. J Ovarian Res 11:1–10 29615083 10.1186/s13048-018-0400-5 PMC5883607

[CR63] Jimoh OA, Ewuola EO, Balogun AS (2017) Oxidative stress markers in exotic breeds of rabbit during peak of heat stress in Ibadan, Nigeria. J Adv Biol Biotechnol 12:1–9

[CR64] Kalia S, Bharti VK, Giri A, Malairaman U, Biswas A, Kumar B (2021) Effect of Salix Alba leave extract on growth performance, antioxidant level and immune status of broiler chickens reared at high altitude cold desert. Bio 76:3003–3015

[CR66] Kettunen H, Peuranen S, Tiihomen K, Saaarinen M (2001) Intestinal uptake of betaine in vitro, and on the distribution of Methyl groups from betaine, choline and methionine in the body of broiler chicks. Comp Biochem Physiol 128A:269–278 10.1016/s1095-6433(00)00301-9 11223388

[CR67] Khan AS (2017) Antipyretic and analgesic activities of some economically important Woody plants. Medicinally important trees. Springer International Publishing, Cham Switzerland, pp 1–309

[CR68] Khan RA, Khan MR, Sahreen S (2012) CCl 4-induced hepatotoxicity: protective effect of Rutin on p53, CYP2E1 and the antioxidative status in rat. BMC Complement Altern Med 12:1–6 10.1186/1472-6882-12-178 PMC3519517 23043521

[CR69] Khodayar MJ, Kalantari H, Khorsandi L, Rashno M, Zeidooni L (2018) Betaine protects mice against acetaminophen hepatotoxicity possibly via mitochondrial complex II and glutathione availability. Biomed Pharmacother 103:1436–1445 29864928 10.1016/j.biopha.2018.04.154

[CR70] Lakhani P, Kumar P, Lakhani N, Bhimte A (2019) Effects of dietary betaine supplementation on growth performance, immunity and oxidative stress in Karan Fries heifers during heat stress. J Chem Stud 7(4):533–541

[CR71] Leslie EM, Mao Q, Oleschuk CJ, Deeley RG, Cole SP (2001) Modulation of multidrug resistance protein 1 (MRP1/ABCC1) transport and ATPase activities by interaction with dietary flavonoids. Mol Pharmacol 59(5):1171–1180 11306701 10.1124/mol.59.5.1171

[CR72] Lever M, Slow S (2010) The clinical significance of betaine, an osmolyte with a key role in Methyl group metabolism. Clin Biochem 43(9):732–744 20346934 10.1016/j.clinbiochem.2010.03.009

[CR73] Li Z, Pu J, Zeng T, Cai J, Jia G, Zhao H, Liu G, Zeng Q, Luo Y, Tian G (2024) Effects of betaine on growth performance and intestinal health of rabbits fed different digestible energy diets. J Anim Sci Jan 3:102:skae029. 10.1093/jas/skae029 10.1093/jas/skae029 PMC10889737 38290533

[CR74] Liang ZL, Chen F, Park S, Balasubramanian B, Liu WC (2022) Impacts of heat stress on rabbit immune Function, Endocrine, blood biochemical Changes, antioxidant capacity and production Performance, and the potential mitigation strategies of nutritional ntervention. Front Vet Sci 9:906084 35720853 10.3389/fvets.2022.906084 PMC9201964

[CR75] Liu Y, Liu S, Wang H, Su W (2021) Protective effect of caffeic acid on streptozotocin induced gestational diabetes mellitus in rats: possible mechanism. Pak J Zool 53:1045–1052

[CR76] Liu W, Chen L, McClements DJ, Zou Y, Chen G, Jin Z (2024) Vanillin-assisted Preparation of chitosan-betaine stabilized corn starch gel: gel properties and microstructure characteristics. Food Hydrocoll 148:109510. 10.1016/j.foodhyd.2023.109510

[CR77] Mahdi JG (2010) Medicinal potential of willow: a chemical perspective of aspirin discovery. J Saudi Chem Soc 14:317–322

[CR78] Mahdi JG, Mahdi AJ, Bowen ID (2006) The historical analysis of aspirin discovery, its relation to the Willow tree and antiproliferative and anticancer potential. Cell Prolif 39:147–155 16542349 10.1111/j.1365-2184.2006.00377.x PMC6496865

[CR79] Marai IFM, Ayytat MS, Abd el-Monem UM (2001) Growth performance and reproductive trans at first parity of new Zealand white female rabbits as affected by heat stress and its alleviation under Egyptian conditions. Trop Anim Health Prod 33:451–462 11770200 10.1023/a:1012772311177

[CR80] Marklund SL, Holme E, Hellner L (1982) Superoxide dismutase in extracellular fluids. Clin Chim Acta 126:41–51 7172448 10.1016/0009-8981(82)90360-6

[CR81] Martin B (2019) The suitability of willow trees as animal forage and their application in zoological institutions. Master’s thesis, University of Guelph, Canada. https://atrium.lib.uoguelph.ca/items/c14b362f-99a9-44dd-a257-0e0fc190f873

[CR82] Metzler-Zebeli BU, Eklund M, Mosenthin R (2009) Impact of osmoregulatory and Methyl donor functions of betaine on intestinal health and performance in poultry. World’s Poult Sci J 65:419–442

[CR83] Minafra CS, Marques SFF, Stringhini JH, Ulhoa CJ, Rezende CSM, Santos JS et al (2010) Perfil bioquímico do Soro de Frangos de Corte alimentados com Dieta suplementada com alfa-amilase de Cryptococcus flavus e Aspergillus Niger. Revista Brasileira De Zootecnia 39(12):26912696

[CR84] Mizera A, Kuczaj M, Szul A (2019) Impact of the spirulina maxima extract addition to semen extender on bovine sperm quality. Ital J Anim Sci 18(1):601–607

[CR85] Mohammadi T, Hosseinchi Gharehaghaji M (2024) The influence of Rutin and chlorogenic acid on oxidative stress and in vivo fertility: evaluation of the quality and antioxidant status of post thaw semen from Azari water Buffalo bulls. Vet Med Sci 10(5), e31548 10.1002/vms3.1548 PMC11332393 39158970

[CR86] Moore GF, Audrey S, Barker M, Bond L, Bonell C, Hardeman W, Moore L, O’Cathain A, Tinati T, Wight D, Baird J (2015) Process evaluation of complex interventions: medical research Council guidance. BMJ 350:h1258 25791983 10.1136/bmj.h1258 PMC4366184

[CR87] Mourvaki E, Cardinali R, Dal Bosco A, Corazzi L, Castellini C (2010) Effects of flaxseed dietary supplementation on sperm quality and on lipid composition of sperm subfractions and prostatic granules in rabbit. Theriogenology 73(5):629–637 20034660 10.1016/j.theriogenology.2009.10.019

[CR88] Mussatto SI, Ballesteros LF, Martins S, Teixeira JA (2011) Extraction of antioxidant phenolic compounds from spent coffee grounds. Sep Purif Technol 83:173–179

[CR89] Nahrstedt A, Schmidt M, Jäggi R, Metz J, Khayyal MT (2007) Willow bark extract: the contribution of polyphenols to the overall effect. Wien Med Wochenschr 157:348–351 17704985 10.1007/s10354-007-0437-3

[CR90] Naseer Z, Ahmad E, Şahiner HS, Epikmen ET, Fiaz M, Yousuf MR, Khan SA, Serin İ, Ceylan A, Aksoy M (2018) Dietary Quercetin maintains the semen quality in rabbits under summer heat stress. Theriogenology 122:88–93 30243139 10.1016/j.theriogenology.2018.09.009

[CR91] Naseer Z, Ahmad E, Aksoy M, Epikmen E (2020) Impact of Quercetin supplementation on testicular functions in summer heat-stressed rabbits. World Rabbit Sci 28(1):19–27

[CR92] Nauman M, Kale RK, Singh RP (2018) Polyphenols of Salix aegyptiaca modulate the activities of drug metabolizing and antioxidant enzymes, and level of lipid peroxidation. BMC Complement Altern Med 18:81 29514630 10.1186/s12906-018-2143-7 PMC5842599

[CR93] Okab A, Koriem A (2008) Inflence of environmental temperatures on some physiological and biochemical parameters of newzealand rabbit males. Slovak J Anim Sci 41:12–19

[CR94] Oladimeji AM, Johnson TG, Metwally K, Farghly M, Mahrose KM (2022) Environmental heat stress in rabbits: implications and ameliorations. Int J Biometeorol 1–11 10.1007/s00484-021-02191-0 34518931

[CR95] Olaniyan MF, Atibor RA, Afolabi T (2018) Evaluation of tumor necrosis factor alpha (TNFα), Interleukin 4, Interleukin 6, aspartate aminotransferase, and Alanine aminotransferase in rabbits overdosed with ibuprofen and supplemented with guava leaf (Psidium guajava) extract. BBRJ 2(4):254–259

[CR96] Olasehinde O, Osawe S, Alilonu D, Shedrack O, Obimma J, Ebokaiwe A (2025) Rutin impedes indoleamine 2, 3-dioxygenase activity/expression to mitigate heat stress-mediated testicular dysfunction. Pharmacol Res Nat Prod 6:100186

[CR97] Oriani G, Corino C, Pastorelli G, Pantaleo L, Ritieni A, Salvatori G (2001) Oxidative status of plasma and muscle in rabbits supplemented with dietary vitamin E. J Nutr Biochem 12(3):138–143. 10.1016/s0955-2863(00)00132-7 11257462 10.1016/s0955-2863(00)00132-7

[CR98] Panaite TD, Saracila M, Papuc CP, Predescu CN, Soica C (2020) Influence of dietary supplementation of Salix Alba bark on performance, oxidative stress parameters in liver and gut microflora of broilers. Animals 10:958 32486449 10.3390/ani10060958 PMC7341264

[CR99] Ratriyanto A, Mosenthin R (2018) Osmoregulatory function of betaine in alleviating heat stress in poultry. J Anim Physiol Anim Nutr 102:1634–1650 10.1111/jpn.12990 30238641

[CR100] Rokade JJ, Saxena VK, Marappan G, Bhanja SK, Chaudhary SK, Kolluri G, Madheswaran M (2020) Effect of dietary betaine supplementation on egg quality, semen quality, hematology, fertility and hatchability in broiler breeders. Indian J Anim Sci 90(7):1024–1029

[CR104] Saracila M, Panaite TD, Vlaicu PA, Tabuc C, Palade ML, Gavris T, Criste RD (2018) Dietary Willow bark extract for broilers reared under heat stress. J Anim Sci Biotechnol 75:92–98

[CR103] Saracila M, Panaite TD, Soica C, Tabuc C, Olteanu M, Predescu C, Rotar CM, Criste RD (2019) Use of a hydroalcoholic extract of Salix Alba L. bark powder in diets of broilers exposed to high heat stress. S Afr J Sci 49:942–954

[CR102] Saracila M, Panaite TD, Papuc CP, Criste RD (2021) Heat stress in broiler chickens and the effect of dietary Polyphenols, with special reference to Willow (Salix spp.) bark Supplements- A review. Antioxidants 10:686 33925609 10.3390/antiox10050686 PMC8146860

[CR105] Seddiki Y, da Silva HM, da Silva FM (2017) Antioxidant properties of polyphenols and their potential use in improvement of male fertility: a review. Biomed J Sci Tech Res 1(3):612–617

[CR106] Shadmehr S, Tabatabaei SRF, Hosseinifar S, Tabandeh MR, Amiri A (2018) Attenuation of heat stress-induced spermatogenesis complications by betaine in mice. Theriogenology 106:117–126 29049923 10.1016/j.theriogenology.2017.10.008

[CR107] Sharma S, Sahu D, Das HR, Sharma D (2011) Amelioration of collagen-induced arthritis by Salix Nigra bark extract via suppression of pro-inflammatory cytokines and oxidative stress. Food Chem Toxicol 49:3395–3406 21983485 10.1016/j.fct.2011.08.013

[CR108] Smith JT, Mayer DT (1955) Evaluation of spermconcentration by the hemacytometer method.Comparison of four counting fluids. Fertil Steril 6:271–275 14380385 10.1016/s0015-0282(16)31987-2

[CR109] Sultana JR, Chandra AS, Ramana D, Raghunandan T, Prakash MG, Venkateswarlu M (2022) Effect of dietary chromium, vitamin E and selenium supplementation on biochemical and physiological parameters of Holstein Friesian cows under heat stress. Indian J Anim Sci 92:858–864

[CR110] Tan BL, Norhaizan ME, Liew WPP, Sulaiman RH (2018) Antioxidant and oxidative stress: A mutual interplay in age-related diseases. Front Pharmacol 9:1162 30405405 10.3389/fphar.2018.01162 PMC6204759

[CR112] Theau-Clément M, Maertens L, Castellini C, Besenfelder U, Boiti C (2005) Recommendations and guidelines for applied reproduction trials with rabbit does. World Rabbit Sci 13(3)

[CR111] Theau-Clément M, Ailloud E, Sanchez A, Saleil G, Brun JM (2016) Relationships between rabbit semen characteristics and fertilising ability after insemination. Animal 10(3):426–431 26549861 10.1017/S1751731115002372

[CR113] Uyanga VA, Oke EO, Amevor FK, Zhao J, Wang X, Jiao H, Lin H (2022) Functional roles of taurine, L-theanine, L-citrulline, and betaine during heat stress in poultry. J Anim Sci Biotechnol 13(1):23 35264238 10.1186/s40104-022-00675-6 PMC8908636

[CR114] Velderrain-Rodríguez GR, Torres-Moreno H, Villegas-Ochoa MA, Ayala-Zavala JF, Robles-Zepeda RE, Wall-Medrano A, González-Aguilar GA (2018) Gallic acid content and an antioxidant mechanism are responsible for the antiproliferative activity of ‘Ataulfo’mango Peel on LS180 cells. Molecules 23(3):695 29562699 10.3390/molecules23030695 PMC6017175

[CR115] Vlachojannis J, Magora F, Chrubasik S (2011) Willow species and aspirin: different mechanism of actions. Phytother Res 25:1102–1104 21226125 10.1002/ptr.3386

[CR116] Walker WH (2009) Molecular mechanisms of testosterone action in spermatogenesis. Steroids 74(7):602–607 19095000 10.1016/j.steroids.2008.11.017

[CR117] Wang H, Li S, Xu S, Feng J (2020) Betaine improves growth performance by increasing digestive enzymes activities, and enhancing intestinal structure of weaned piglets. Anim Feed Sci Technol 267:114545

[CR118] Wen C, Leng Z, Chen Y, Ding L, Wang T, Zhou Y (2021) Betaine alleviates heat stress-induced hepatic and mitochondrial oxidative damage in broilers. J Poult Sci 58(2):103–109 33927564 10.2141/jpsa.0200003 PMC8076623

[CR119] Yao Z, Vance D (1989) Head group specificity in the requirement of phosphatidylcholine biosynthesis for very low density lipoprotein secretion from cultured hepatocytes. J Biol Chem 264:11373–11380 2738069

[CR120] Yousef MS, Megahed GA, Abozed GF, Hayder M, Plo HH, Rawy MS (2022) Exogenous gonadotropin-releasing hormone counteracts the adverse effect of scrotal insulation on testicular functions in bucks. Sci Rep 12:7869 35551262 10.1038/s41598-022-11884-4 PMC9098548

[CR121] Zabihi AN, Mahmoudabady M, Soukhtanloo M, Hayatdavoudi P, Beheshti F, Niazmand S (2018) Salix Alba attenuated oxidative stress in the heart and kidney of hypercholesterolemic rabbits. Avicenna J Phytomed 8:63–72 29379769 PMC5784080

[CR122] Zern TL, Fernandez ML (2005) Cardioprotective effects of dietary polyphenols. J Nutr 135(10):2291–2294 16177184 10.1093/jn/135.10.2291

[CR125] Zhang X (2011) Application of total bile acid, ALT and AST in serum. Jilin Med J 32:4840–4841

[CR123] Zhang L, Ying SJ, An WJ, Lian H, Zhou GB, Han ZY (2014) Effects of dietary betaine supplementation subjected to heat stress on milk performances and physiology indices in dairy cow. Genet Mol Res 13(3):7577–7586 25222258 10.4238/2014.September.12.25

[CR124] Zhang M, Zhang H, Li H, Lai F, Li X, Tang Y, Min T, Wu H (2016) Antioxidant mechanism of betaine without free radical scavenging ability. J Agric Food Chem 64(42):7921–7930. 10.1021/acs.jafc.6b03592 27677203 10.1021/acs.jafc.6b03592

[CR126] Zhao G, He F, Wu C, Li P, Li N, Deng J, Zhu G, Ren W, Peng Y (2018) Betaine in Inflammation: Mechanistic Aspects and Applications. Front Immunol 2018; 9:1070. 10.3389/fimmu.2018.01070 10.3389/fimmu.2018.01070 PMC5976740 29881379

